# Combining radiomics and deep learning to predict liver metastasis of gastric cancer on CT image

**DOI:** 10.3389/fonc.2025.1613972

**Published:** 2025-06-24

**Authors:** Yimin Guo, Haixiang Yin, Hanyue Zhang, Pan Liang, Jianbo Gao, Ming Cheng

**Affiliations:** ^1^ Department of Radiology, The First Affiliated Hospital of Zhengzhou University, Zhengzhou, Henan, China; ^2^ Henan Key Laboratory of Image Diagnosis and Treatment for Digestive System Tumor, The First Affiliated Hospital of Zhengzhou University, Zhengzhou, Henan, China; ^3^ Department of Medical Information, The First Affiliated Hospital of Zhengzhou University, Zhengzhou, Henan, China; ^4^ Institute of Interconnected Intelligent Health Management of Henan Province, The First Affiliated Hospital of Zhengzhou University, Zhengzhou, Henan, China

**Keywords:** deep learning, radiomics nomogram, gastric cancer, liver metastasis, computed tomography

## Abstract

**Objective:**

Our study aimed to explore the potential of deep learning (DL) radiomics features from CT images of primary gastric cancer (GC) in predicting gastric cancer liver metastasis (GCLM) by establishing and verifying a prediction model based on clinical factors, classical radiomics and DL features.

**Methods:**

We retrospectively analyzed 1001 pathologically confirmed GC patients from June 2014 to May 2024, divided into non-LM (n=689) and LM groups (n=312). CT-based classic radiomics and DL features were extracted and screened to construct a DL-radiomics score. This score, along with statistically significant clinical factors, was used to build a fused model which visualized as a nomogram. The model’s predictive performance, calibration, and clinical utility were assessed and compared against a clinical model. Additionally, the DL-radiomics score’s role in distinguishing between synchronous and metachronous GCLM was evaluated.

**Results:**

The fused model showed good predictive performance [AUC: 0.796 (95% CI: 0.766-0.826) in training cohort and 0.787 (95% CI: 0.741-0.834) in test cohort], outperforming the clinical model, radiomics score and DL score (P<0.05). In addition, the decision curve confirmed that the model provided the largest clinical net benefit compared with all other models in the relevant threshold. DL-radiomics score showed moderate predictive performance in distinguishing between synchronous GCLM and metachronous GCLM, with an AUC of 0.665 (95% CI, 0.613-0.718).

**Conclusion:**

The CT-based fused model has demonstrated significant value in predicting the occurrence of GCLM, and can provide a reference for the personalized follow-up and treatment of patients.

## 1 Introduction

As the fifth most common cancer diagnosis and the leading cause of cancer-related death in the world, poor prognosis of gastric cancer (GC) poses a serious challenge to human health (1). One primary contributor to this adverse outcome is the tendency of GC to metastasize distantly with common sites include liver, peritoneum, and bone (2). Among these, the liver stands out as the primary target organ for hematogenous metastasis of GC. The overall incidence of gastric cancer liver metastases (GCLM) is about 9.9%~18.7%, of which synchronous GCLM accounts for about 80% and metachronous GCLM accounts for about 20%. A number of studies have shown that the overall survival of patients with synchronous GCLM is worse than that of patients with metachronous GCLM (3).

At present, the predominant imaging methods employed for detecting liver metastasis (LM) in patients with GC are computed tomography (CT) and magnetic resonance imaging (MRI). Among them, CT is more widely used due to its reasonable price, convenience and less contraindications (4). However, it is difficult to detect early micro-lesions or micro-metastasis by traditional CT, and the LM after GC surgery, i.e., metachronous GCLM, cannot be predicted early, which may lead to missing the best time for treatment (5). The accuracy and sensitivity of MRI in the diagnosis of LM are higher than CT. However, due to its high price and long examination time, it is usually used as a supplementary test in clinical practice only when other examination methods have found suspicious LM. Based on the above reasons, it is particularly important to find a CT-based method to predict the occurrence of GCLM and screen out high-risk patients with GCLM.

In recent years, artificial intelligence has gradually penetrated into the field of medical research, especially in medical imaging, so radiomics came into being (6). It can be used as a non-invasive visualization tool to extract tumor features and reveal tumor heterogeneity. Classical radiomics feature extraction relies on predefined statistical descriptors, such as shape, pixel intensity, and texture. Some scholars have applied this method to the prediction of colon cancer liver metastases (CCLM), and achieved good results, which confirmed the feasibility of this method (7–10). Deep learning (DL), based on deep neural networks, can automatically learn and extract valuable features from original medical images without pre-definition (11–13). The features extracted by the two methods reflect abstract information at different levels of tumor imaging, and reveal the imaging features of tumors more comprehensively. To the best of our knowledge, existing studies predicting the occurrence of GCLM have relied solely on clinical characteristics or visually assessable CT image features (4, 5, 14, 15) and no research has used mixing of classic radiomics features and DL features to predict the occurrence of GCLM, and this study may be the first attempt.

Our study aimed to explore the potential of radiomics features from CT images of primary GC in predicting GCLM, and to establish and verify a prediction model based on clinical factors, classical radiomics features and DL features. In addition, we further evaluated the ability of the DL-radiomics score constructed by the selected classical radiomics features and DL features to distinguish between synchronous GCLM and metachronous GCLM.

## 2 Materials and methods

### 2.1 Patients

The Institutional Review Board of the First Affiliated Hospital of Zhengzhou University approved our research (Ethical review number: 2021-KY-1070-002) and waived the requirement of written informed consent. We retrospectively collected patients with GC confirmed by pathology in the First Affiliated Hospital of Zhengzhou University from June 2014 to May 2024, and screened patients based on the following inclusion and exclusion criteria.

The inclusion criteria were:

Non-LM Group: (1) Patients were followed up regularly for at least two years in our hospital and there was no evidence of liver metastasis during the follow-up period; (2) Patients had complete clinical data.

LM Group: (1) Patients found liver metastases that could be confirmed by pathology or imaging examination during the follow-up period; (2) Patients had complete clinical data.

And the common exclusion criteria were: (1) The patient had primary malignant tumors in other organs; (2) The patient had a history of gastric cancer treatment; (3) CT image quality is poor or insufficient stomach distension.

**Figure 1:**
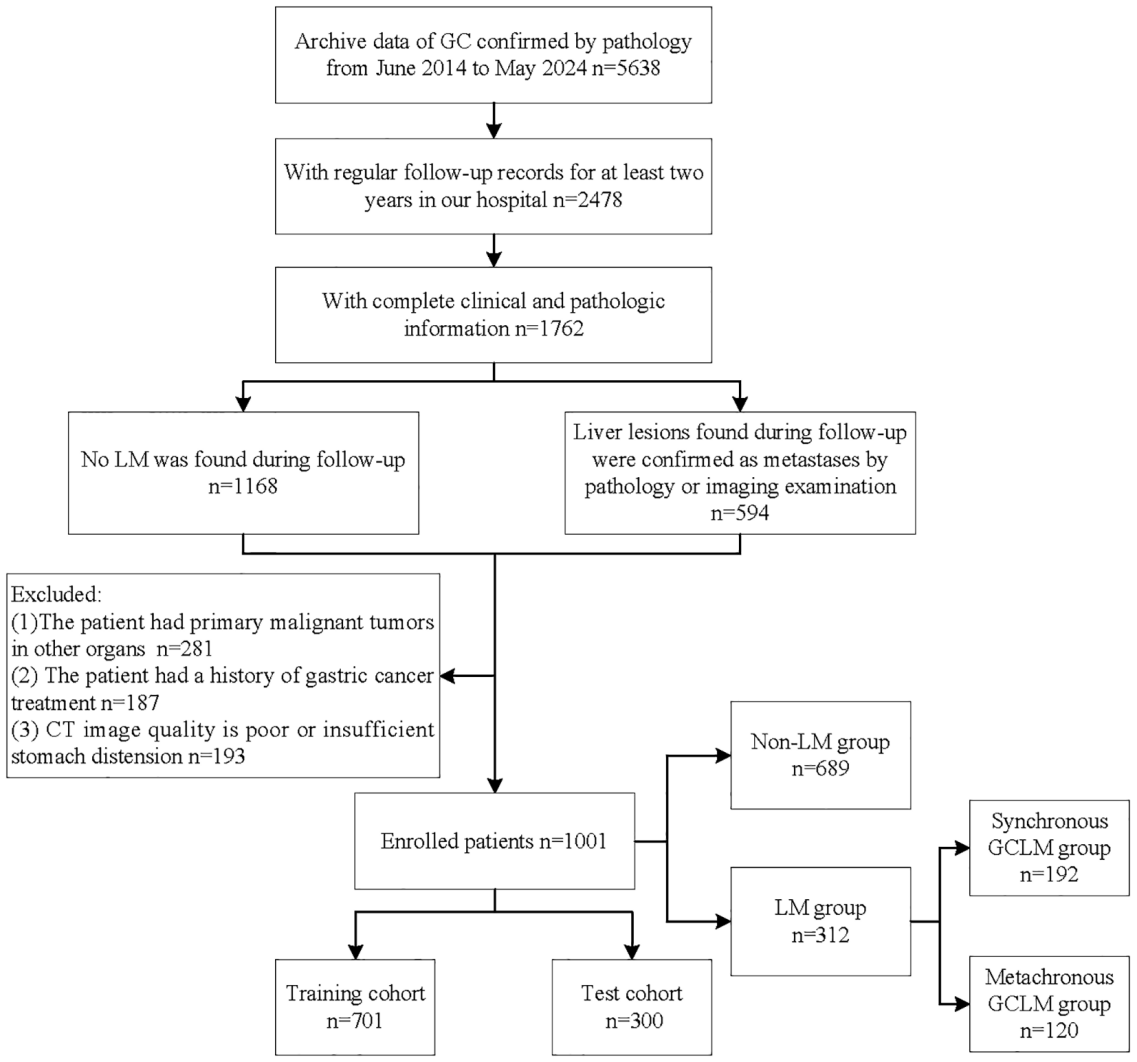
Flowchart of patient selection process. GC, gastric cancer; LM, liver metastases; GCLM, gastric cancer liver metastases.

Finally, we included a total of 1001 patients. According to whether the patients had LM during the follow-up period, we divided them into two groups, including non-LM group (n=689) and LM group (n=312). A flowchart detailing the procedure of patient selection is displayed in **Figure 1**. Baseline clinical information of patients was collected, including sex, age, tumor location, tumor thickness, clinical T stage, clinical N stage, degree of differentiation, Lauren type, Her-2 lever, CEA, CA199. Based on the computer-generated random numbers, patients were randomly divided into the training cohort and the test cohort at a ratio of 7: 3.

### 2.2 CT image acquisition and image preprocessing

All patients underwent abdominal enhanced CT examination before receiving GC treatment. The venous phase cross-sectional CT image with a thickness of 5 mm was selected to delineate the region of interest (ROI). The CT examination method is shown in detail in the **Supplemetary Appendix S1**. A radiologist (Reader 1), with over eight years of experience in interpreting medical films, utilized 3D Slicer software to outline ROI along the tumor’s edge in all CT scans capable of displaying gastric malignancies. After a month, Reader 1 was reassigned, and a second radiologist (Reader 2), also possessing more than eight years of experience, was chosen for the task. CT images from 100 patients diagnosed with GC were randomly chosen from the study cohort. Both Reader 1 and Reader 2 then independently repeated the segmentation of ROIs to assess intra- and inter-observer reproducibility. Intraclass and interclass correlation coefficients (ICCs) were computed to quantify agreement. Reliability was deemed satisfactory when ICC values exceeded 0.8.

Before segmentation and feature extraction, image preprocessing is performed to improve the stability of radiological features. In order to standardize CT images from different CT scanning equipment, two steps are used: (a) all CT images were resampled to a voxel size of 1× 1 × 1 mm³ using cubic spline interpolation; and (b) the pixel intensity was normalized to transform the images to standardized inputs, which had the intensity range from -1024 to 1024 HU and the unified abdomen window (window-level [WL] of 50 and window-width [WW] of 350).

### 2.3 DL radiomics features extraction

An autoencoder (AE), constructed based on a deep convolutional neural network (DCNN), was employed to DL features. The AE comprised two primary components: a 3D encoder and a 3D decoder. The 3D encoder functioned to automatically extract latent-space vectors from 3D ROI. Subsequently, the decoder reconstructed CT slices from these latent-space vectors, ensuring that the reconstructed slices closely matched the original input to the encoder. In this study, these latent-space vectors were referred to as DL features. In addition, classical radiomics features were extracted using Pyradiomics (http://pyradiomics.readthedocs.io) and included shape features, first-order features, second-order features, high-order features. All the feature extraction methods were further explained in the **Supplemetary Appendix S2**.

### 2.4 Feature selection and model construction

The feature screening process was carried out according to the following steps in the training cohort. Firstly, we applied the variance threshold method to filter out features with a variance not exceeding 1.0. Then, Spearman correlation analysis was performed to remove features that had an average correlation coefficient greater than 0.7. Next, we used the independent sample t-test to select features that exhibited significant differences (*P* < 0.05) with the target variable. Ultimately, the least absolute shrinkage and selection operator (LASSO) algorithm was applied to was utilized to further refine the selection of features highly correlated with GCLM. LASSO regression operates by introducing an L1 regularization term with parameter λ to the loss function, so that the weights of irrelevant features are 0, thereby achieving feature selection and preventing overfitting. We performed a 10-fold cross-validation to determine the optimal λ value, and the optimal λ defined as the largest value within one standard error of the minimum binomial deviance. Consequently, multivariable logistic regression analysis was used to build two types of scores, radiomics and DL, reflecting the different phenotypic characteristics of the tumors. The DL-radiomics score combining the DL and classical radiomics features were also constructed.

**Figure 2:**
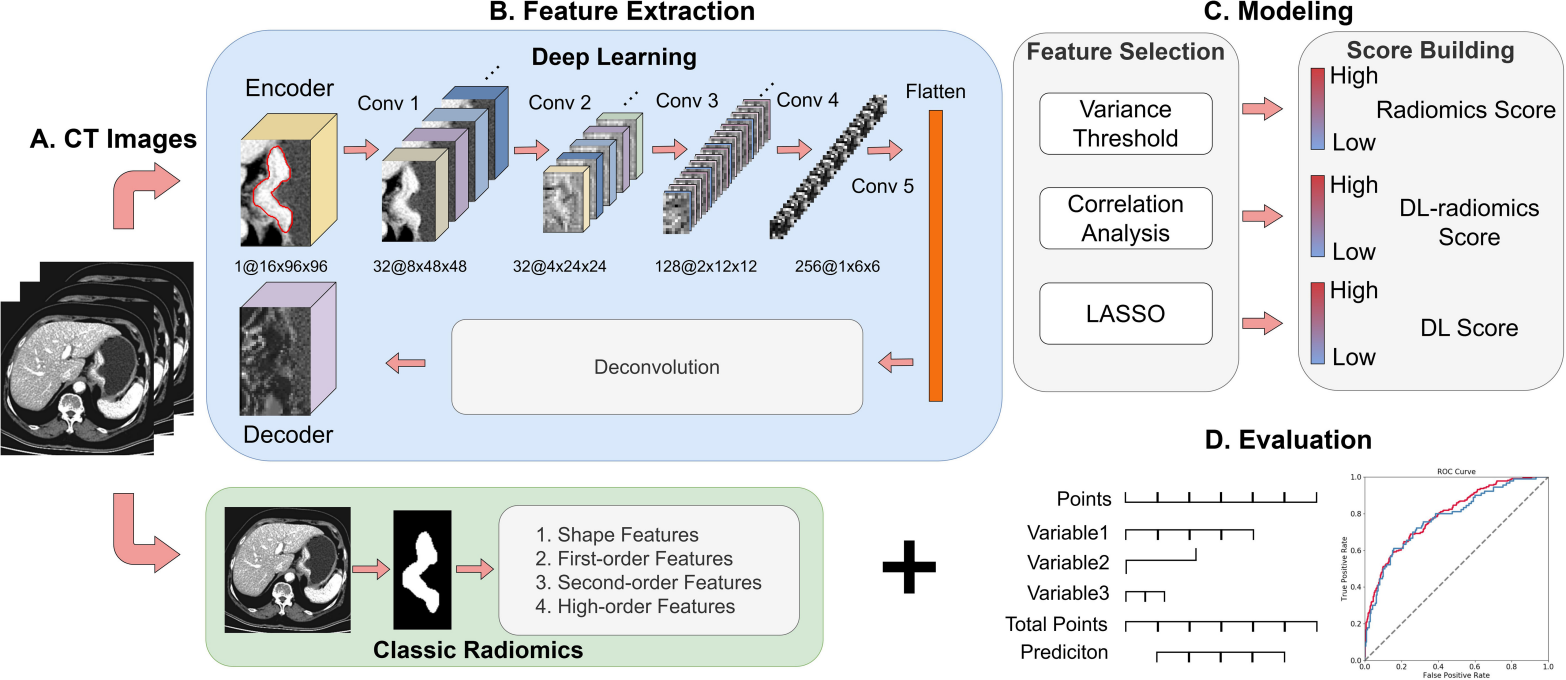
Overview of the study design. **(A)** Collection of 5mm venous phase CT Images; **(B)** Extraction of DL features and classical radiomics features; (C) Feature selection and model construction; **(D)** Model visualization and evaluation. DL, deep learning.

Univariable analysis was performed to identify statistically significant clinical factors (*P* < 0.05). Subsequently, multivariable logistic regression analysis was employed to develop a fused model by combining the DL-radiomics score and the significant clinical factors. Then, a fused nomogram was generated to provide the clinician with an applicable tool to estimate the probability of future LM in patients with GC. The model construction process is shown in **Figure 2**. Additionally, a clinical model containing only clinical variables was built for comparison.

### 2.5 Performance evaluation

The receiver operator characteristic (ROC) curves of each model were drawn respectively to obtain the area under the curve (AUC) value and 95% confidence interval (CI). Then DeLong ‘s test was used to compare whether the differences between different ROC curves were statistically significant. Then we applied the confused matrix to further evaluate other performance indicators of the models, including accuracy (ACC), sensitivity (SENS), specificity (SPEC), positive predictive value (PPV), and negative predictive value (NPV). In addition, we plotted the calibration curves to evaluate the consistency between the predicted probability of the models and the actual probability. The decision curve was used to evaluate the clinical application value of the models, thereby judging the net benefit of the models in practical application.

We collected the interval time of LM patients from the diagnosis of GC to the diagnosis of LM. Patients with an interval time of less than 6 months were included in the synchronous GCLM group (n=192), while those with an interval time exceeding 6 months were placed in the metachronous GCLM group (n=120). We further explored the ability of the DL-radiomics score to distinguish patients with synchronous GCLM from patients with metachronous GCLM.

### 2.6 Statistical analysis

We used Python 3.6, R software 4.0.3 (R project for statistical computing, https://www.r-project.org) to analyze baseline clinical information. Categorical variables were manifested as numbers or percentages, and Chi-square analysis was performed to analyze categorical data. Means and SDs were used to present continuous variables. Differences between the two groups were assessed using t-tests if the data conformed to a normal distribution and had equal variance; otherwise, Mann-Whitney U tests were applied. Statistical significance was set at *P* < 0.05.

## 3 Results

### 3.1 Patient characteristics

The clinical information of the 1001 patients (77.82% males; mean age, 59.46 ± 10.40; range, 20–88 years) we finally included is shown in **Supplementary Table S1**. According to the ratio of 7:3, we randomly divided all samples into training cohort (n=701, 79.50% males; mean age, 59.60 ± 10.35; range, 20–86 years) and test cohort (n=300, 74.00% males; mean age, 59.12 ± 10.54; range, 23–88 years). There was no significant difference in clinical characteristics between the training cohort and the test cohort (**Supplementary Table S2**, *P*>0.05). In addition, the clinical characteristics of non-LM group and LM group in different cohorts were compared and the detailed results are shown in **Table 1**. The results revealed tumor thickness, CEA and CA199 showed significant variations in training cohort and test cohort between non-LM group and LM group (*P*<0.05).

**Table 1:** The clinical characteristics of patients in the training and test cohorts.

Characteristics	Training cohort (n=701)			Test cohort (n=300)		
	Non-LM(n=475)	LM(n=226)	P	Non-LM(n=214)	LM(n=86)	P
Age (mean ± SD, years)	58.91±10.15	61.06±10.63	0.010*	59.40±10.62	60.92±10.18	0.061
Sex, No. (%)			0.074			0.405
Female	107(22.5)	37(16.4)		59(27.6)	19(22.1)	
Male	368 (77.5)	189(83.6)		155(72.4)	67(77.9)	
Tumor location, No. (%)			0.012*			0.475
Cardia/fundus	181 (38.1)	61 (27.0)		75(35.1)	32(37.2)	
Body	81 (17.0)	44 (19.5)		40(18.7)	15(17.4)	
Antrum	119 (25.1)	57 (25.2)		60(28.0)	18(20.9)	
More than two-thirds of stomach	94 (19.8)	64 (28.3)		39(15.2)	21(24.5)	
Tumor thickness [mean ± SD, (mm)]	16.79±5.81	19.15±7.41	＜0.001*	16.89±7.36	20.46±7.47	＜0.001*
Clinical T stage, No. (%)			0.585			0.079
T1	34(7.2)	19(8.4)		28(13.1)	4(4.6)	
T2	85(17.9)	32(14.2)		39(18.2)	9(10.5)	
T3	219(46.1)	104(46.0)		98(45.8)	43(50.0)	
T4	137(28.8)	71(31.4)		49(22.9)	30(34.9)	
Clinical N stage, No. (%)			＜0.001*			0.086
N0	151(31.8)	46(20.3)		78(36.4)	15(17.5)	
N1	90(18.9)	64(28.3)		38(17.8)	21(24.4)	
N2	115(24.2)	72(31.9)		41(19.2)	27(31.4)	
N3	119(25.1)	44(19.5)		57(26.6)	23(26.7)	
Degree of differentiation, No. (%)			0.228			0.597
Un-/poorly differentiated	255(53.7)	133(58.8)		118(55.1)	33(38.3)	
Moderately/highly differentiated	220(46.3)	93(41.2)		96(44.9)	35(40.7)	
Lauren type, No. (%)			0.345			0.062
Intestinal	175(36.8)	84(37.2)		68(31.8)	43(50.0)	
Diffuse	151(31.8)	82(36.3)		70(32.7)	22(25.6)	
Mixed	149(31.4)	60(26.5)		76(35.5)	21(24.4)	
Her-2 lever, No. (%)			0.167			0.948
Negative	217(45.7)	90(39.8)		82(38.3)	34(39.5)	
Positive	258(54.3)	136(60.2)		132(61.7)	52(60.5)	
CEA, No. (%)			＜0.001*			＜0.001*
≤5(Normal)	385(81.1)	133(58.8)		174(81.3)	43(50.0)	
>5(Abnormal)	90(18.9)	93(41.2)		40(18.7)	43(50.0)	
CA199, No. (%)			＜0.001*			＜0.001*
≤37(Normal)	411(86.5)	155(68.6)		182(85.0)	56(65.1)	
>37(Abnormal)	64(13.5)	71(31.4)		32(15.0)	30(34.9)	

LM, liver metastasis; CEA, carcinoembryonic antigen; CA199, Carbohydrate antigen199; *p<0.05.

**Figure 3:**
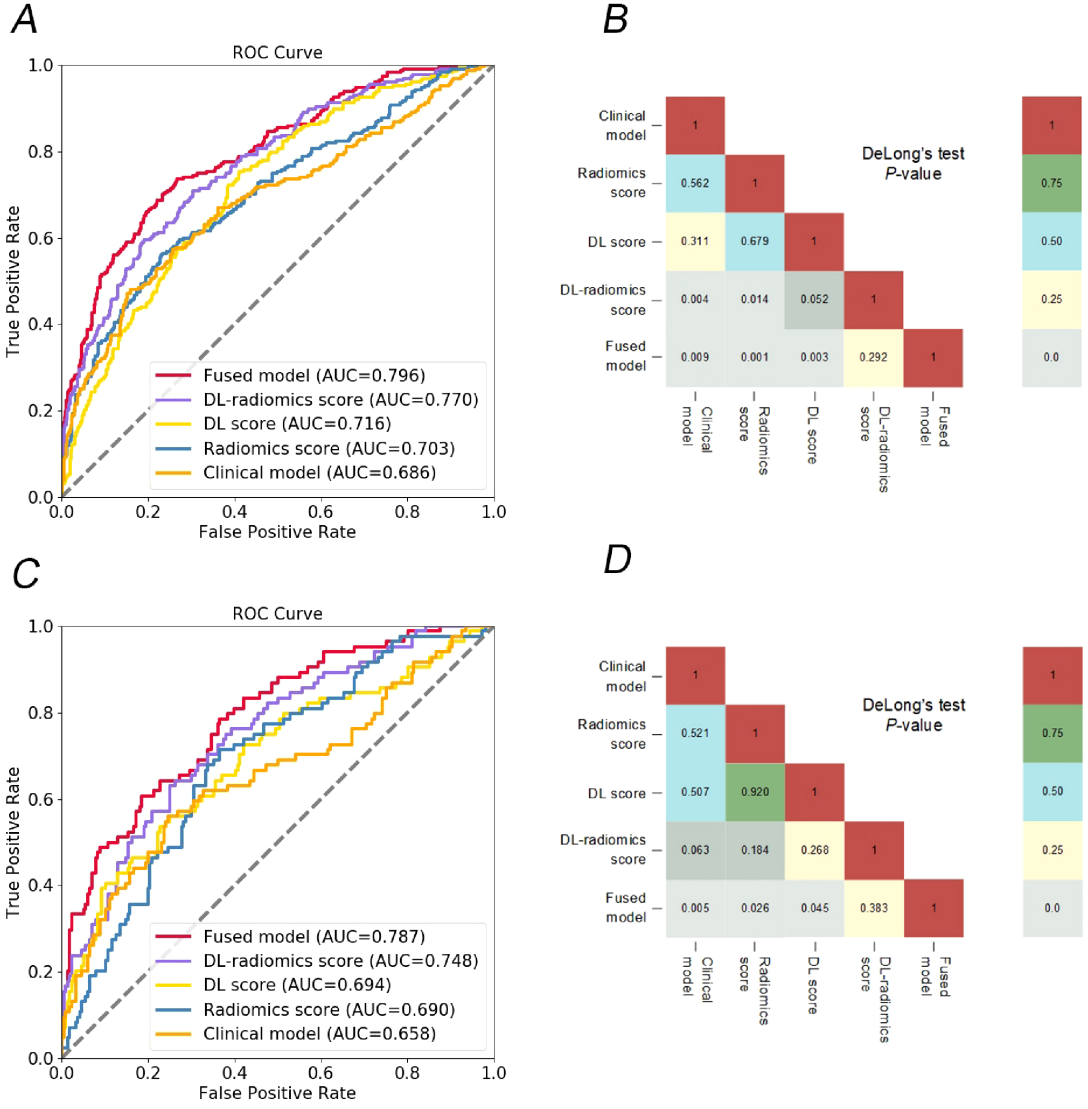
Comparison of different models. ROC curves of different models to predict the occurrence of GCLM, in training cohort **(A)** and test cohort **(C)**; the heat map shows that the DeLong test compares the statistical results of the AUC values of different models, in training cohort **(B)** and test cohort **(D)**. DL, deep learning; ROC, receiver operator characteristic; GCLM, gastric cancer liver metastases.

Then, tumor thickness, CEA and CA199 were used to construct a clinical model. ROC curves of the model were plotted in the two cohorts (**Figures 3A, C**). The AUCs were 0.686 (95% CI, 0.652-0.721) in the training cohort and 0.658 (95% CI, 0.605-0.712) in the test cohort, respectively, showing a moderate ability to predict the occurrence of GCLM. The results are specifically outlined in **Table 2**.

**Table 2:** Performance of different models.

Cohort	Model	AUC	ACC	SENS	SPEC	PPV	NPV
Training cohort	Clinical model	.686 (.652,.721)	.723 (.690, .756)	.476 (.411, .541)	.841 (.809, .874)	.590 (.519, .661)	.770 (.734, .806)
	Radiomics score	.703 (.669, .737)	.700 (.666, .734)	.559 (.495, .624)	.767 (.729, .806)	.536 (.472, .599)	.784 (.747, .822)
	DL score	.716 (.683, .75)	.640 (.604, .676)	.749 (.692,.805)	.588 (.543, .632)	.466 (.415, .517)	.830 (.790,.870)
	DL-radiomics score	.770 (.739, .801)	.699 (.665, .733)	.705 (.646, .764)	.696 (.654, .737)	.526 (.470,.582)	.831 (.794, .868)
	Fused model	.796 (.766, .826)	.733 (.700,.766)	.731 (.674,.789)	.734 (.694,.773)	.568 (.512,.625)	.850 (.816, .885)
Test cohort	Clinical model	.658 (.605, .712)	.703 (.652, .755)	.452 (.346, .559)	.801 (.748, .854)	.469 (.360,.578)	790 (.736, .844)
	Radiomics score	.690 (.638, .742)	.607 (.551, .662)	.750 (.657, .843)	.551 (.485, .617)	.394 (.318, .469)	.850 (.791, .909)
	DL score	.694 (.642, .746)	.703 (.652, .755)	.536 (.429, .642)	.769 (.712, .825)	.474 (.373, .574)	.810 (.756, .863)
	DL-radiomics score	.748 (.699, .797)	.663 (.610,.717)	.702 (.605,.800)	.648 (.584, .712)	.437 (.353, .521)	.848 (.794, .903)
	Fused model	.787 (.741, .834)	.683 (.631, .736)	.679 (.579, .778)	.685 (.623, .747)	.456 (.369, .543)	.846 (.792, .899)

AUC, area under the receiver operating characteristic curve; CI, confidence intervals; ACC, accuracy; SENS: sensitivity; SPEC, specificity; PPV, positive predictive value; NPV, negative predictive value.

### 3.2 DL radiomics score construction

**Figure 4:**
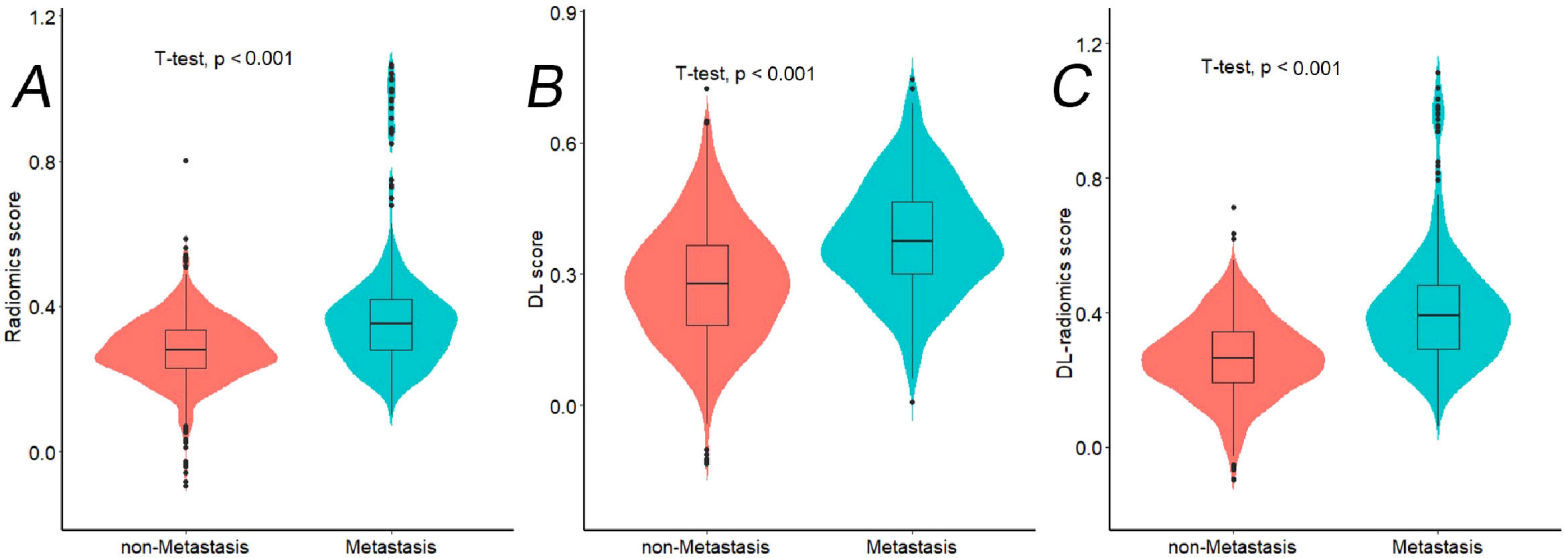
The violin plots showing distribution of different radiomics scores between Non-LM group and LM group in training cohort. **(A)** Radiomics score; **(B)** DL score; **(C)** DL-radiomics score. LM, liver metastases; DL: deep learning; LM: liver metastases.

In the training cohort, we extracted 2437 features from the 3D ROI of GC, including 1925 classic radiomics features and 512 DL features. We screened the features separately to remove irrelevant features and reduce feature redundancy. Finally, 39 classic radiomics and 29 DL features were retained, which were used to construct radiomics and DL score, respectively. Furthermore, same features screening process was conducted on two types of radiomics features, ultimately retaining 57 radiomics features, which was used to construct a DL-radiomics score. Please refer to **Supplemetary Appendix S3** for detailed results of feature screening and scores construction methods. **Figure 4** illustrated that the distribution of different scores between non-LM group and LM group exhibited statistically significant differences, and the score of LM group is generally higher than that of non-LM group (*P*<0.05).

### 3.3 Performance and validation of different models

We evaluated the predictive ability of scores for predicting GCLM. The results showed that in the training cohort, the AUC of the radiomics score, the DL score and the DL-radiomics score were 0.703 (95% CI, 0.669-0.737), 0.716 (95% CI, 0.683-0.75) and 0.770 (95% CI, 0.739-0.801), respectively. In the test cohort, the AUC of the three scores were 0.690 (95% CI, 0.638-0.742), 0.694 (95% CI, 0.642-0.746) and 0.748 (95% CI, 0.699-0.797), respectively. In all cohorts, the AUC of the DL-radiomics score combined with the two types of features both was significantly higher than that of the radiomics score and the DL score, the difference was statistically significant with the DeLong test (*P* < 0.05), indicating that it had better predictive performance (**Table 2**, **Figure 3**).

**Figure 5:**
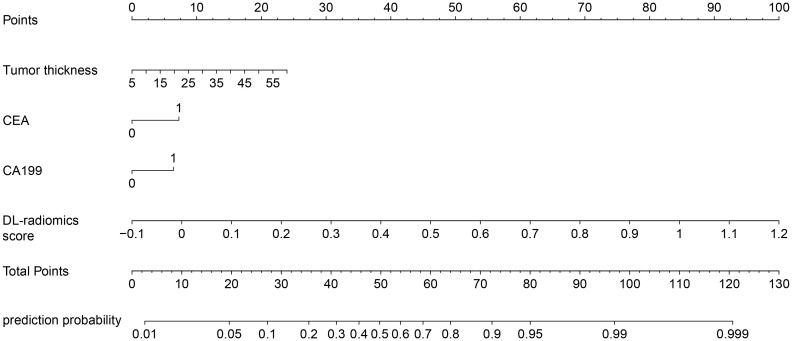
Fused nomogram with the DL-radiomics score and clinical factors (tumor thickness, CEA and CA199). DL, deep learning; CEA, carcinoembryonic antigen; CA199, Carbohydrate antigen199.

**Figure 6:**
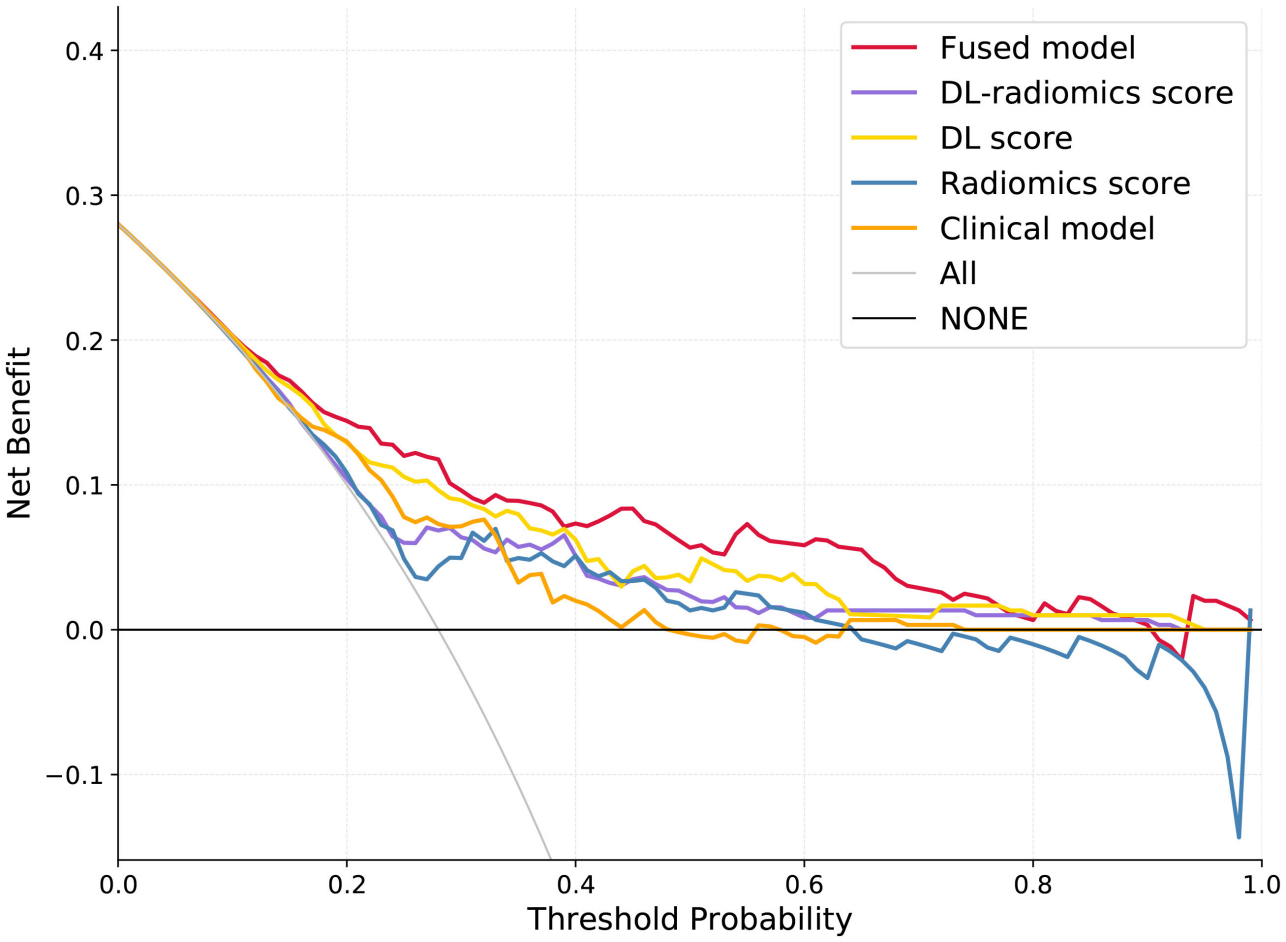
Decision curves analysis for different models. DL, deep learning.

**Figure 7:**
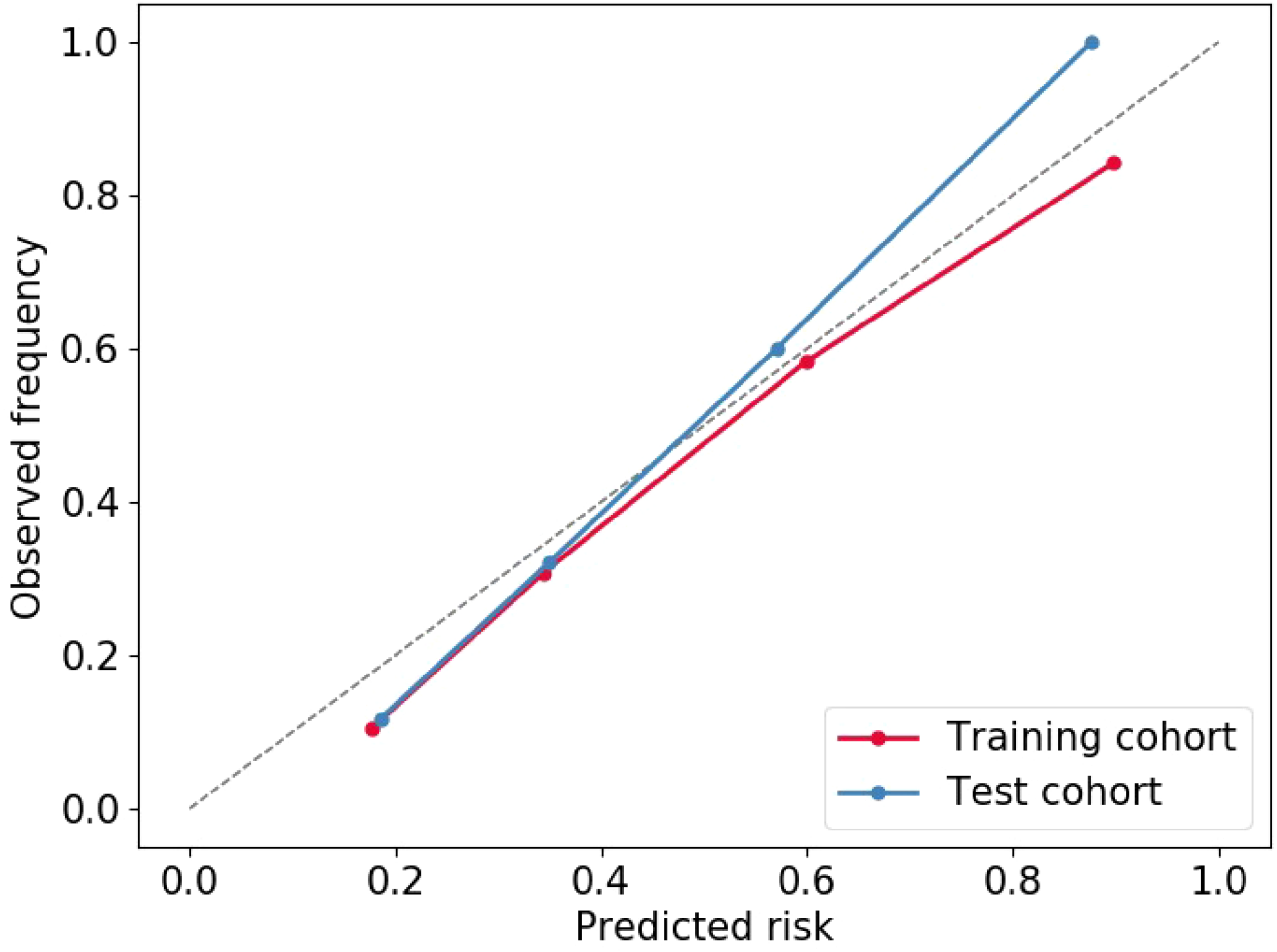
Calibration curve of Fused model to predict the of GCLM occurrence. GCLM, gastric cancer liver metastases.

Multivariable logistic regression analysis results showed that DL-radiomics score and tumor thickness, CEA and CA199 were independent predictors of LM (**Supplementary Table S3**). Therefore, we combine them to construct a fused model and a fused nomogram generated based on fused model was displayed in **Figure 5**. Fused model showed good predictive performance in both cohorts, with AUC values greater than 0.78 [0.796 (95% CI, 0.766-0.826) in the training cohort, 0.787 (95% CI, 0.741-0.834) in the test cohort]. Compared with any other model constructed in our study, the AUC value of the fused model is the highest, which indicated that the fused model has good discrimination between the LM group and the non-LM group. The DeLong test confirmed the AUC value of the fused model was higher than that of other models (*P* < 0.05) except DL-radiomics score (*P* > 0.05), which also indicated that the model combining classical radiomics and DL features achieved better performance than any of them alone (**Table 2**, **Figure 3**). As shown in the decision curve (**Figure 6**), the fused model demonstrated a significant net benefit compared to other models across the relevant threshold range for the whole cohorts. Meanwhile, we observed within almost all threshold ranges, the fused model consistently outperformed both treat-all and treat-none strategies. In addition, the calibration curve showed a good calibration of the fused model, as shown in the **Figure 7**.

### 3.4 Synchronous GCLM and metachronous GCLM

**Figure 8:**
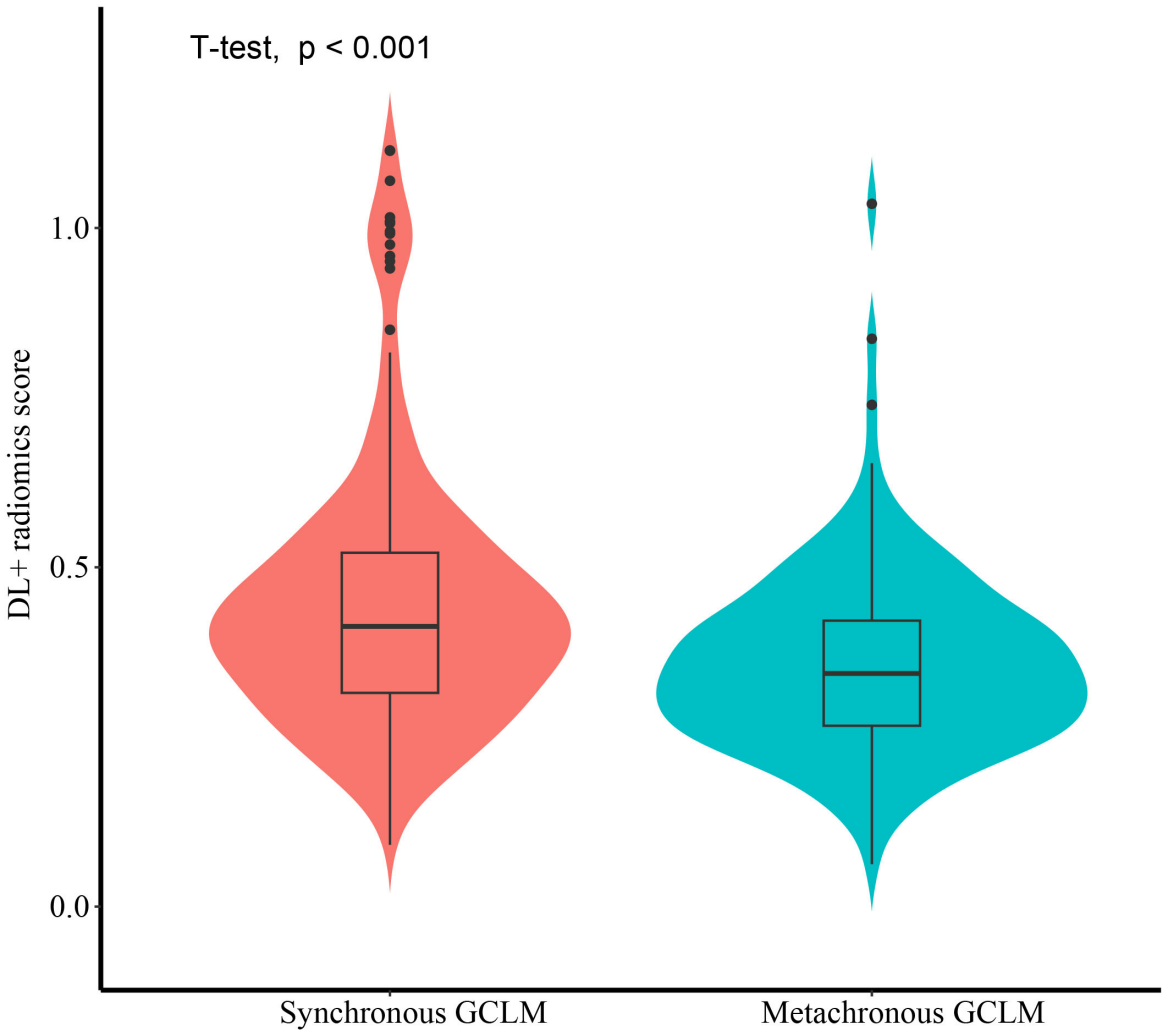
The violin plot illustrating the distribution of DL-Radiomics score for both synchronous GCLM and metachronous GCLM. DL, deep learning; GCLM, gastric cancer liver metastases.

**Figure 8** illustrated the distribution of DL-radiomics score between patients with synchronous GCLM and metachronous GCLM. DL-radiomics scores are higher in patients with synchronous GCLM (*P* < 0.05), indicating that patients with high radiomics scores were more likely to have early LM. The discriminatory capacity of the DL-radiomics score was further evaluated using ROC curves (**Supplementary Figure S1**), with an AUC of 0.665 (95% CI, 0.613-0.718) (**Table 3**), indicating a moderate ability of DL-radiomics score to differentiate between patients with synchronous GCLM and metachronous GCLM. In addition, we also evaluated the ability of other scores to distinguish patients with synchronous GCLM and metachronous GCLM. The detailed results were shown in **Supplementary Table S4** and **Supplementary Figure S1**.

**Table 3:** The performance of DL-radiomics score in distinguishing synchronous GCLM from metachronous GCLM.

Model	AUC	ACC	SENS	SPEC	PPV	NPV
DL-Radiomics score	.665 (.613, .718)	.608 (.553, .662)	.725 (.645, .805)	.534 (.463, .605)	.494 (.420, .568)	.756 (.683, .828)

GCLM, gastric cancer liver metastases; ACC, accuracy; SENS: sensitivity; SPEC, specificity; PPV, positive predictive value; NPV, negative predictive value.

## 4 Discussion

As a stage IV b disease (16), GCLM is one of the important reasons for the poor prognosis of GC. However, CT is less sensitive to detect early micro-metastases of LM and cannot predict metachronous GCLM, which may lead to treatment delay. At present, many scholars have made many attempts to predict the occurrence of GCLM. Yang et al. established and validated a model containing clinical and radiological features to predict LM after resection in patients with GC before surgery (4). Similarly, She et al. retrospectively analyzed the clinical and spectral CT data of 80 patients with GC who underwent surgical resection, and constructed a clinical indicator-spectral CT iodine concentration model to explore its value in predicting GCLM (14). Unlike them, our study integrates classical radiomics and DL features to deeply mine the deep information hidden in CT images, and combines them with patients’ clinical characteristics to establish a fused model for GCLM. This model achieved optimal predictive performance among all models constructed.

We analyzed the baseline clinical information of the two groups of patients, and found that tumor thickness, CEA level and CA199 level were independent predictors of GCLM, which was consistent with some previous related research results (5, 17–19). The occurrence of LM may be caused by the gradual progression of GC. GC progresses from the innermost mucosal layer of the stomach wall outward. As tumor thickness increases, cancer cells are more likely to detach from the gastric wall, leading to an elevated risk of LM (15, 18, 20). Serum tumor markers, such as CEA and CA19-9, serve as valuable indicators for the recurrence or metastasis of gastrointestinal cancers. The elevation of serum tumor markers may precede the detection of abnormalities by imaging examination, thereby aiding clinicians in the earlier identification of diseases or postoperative recurrences (21–23). Similarly, our study found that the abnormal proportion of CEA and CA199 in patients with GCLM was higher than that in patients non-LM, and were independent risk factors for LM.

Radiomics enables the extraction of numerous quantitative features from medical images to describe the heterogeneity, morphology and texture of tumors (24). These features can be used to predict the biological behavior of tumors (25), treatment response (26, 27) and prognosis of patients (28, 29). In our study, image-based features were computed with classical radiomics and DL, respectively, which were then utilized to construct three scores: radiomics score, DL score and DL-radiomics score. Each score demonstrated a certain predictive capacity for GCLM [AUC of radiomics score: 0.690 (95% CI, 0.638-0.742); AUC of DL score: 0.694 (95% CI, 0.642-0.746) and AUC of DL-radiomics score: 0.748 (95% CI, 0.699-0.797)]. Among them, the performance of the DL score is comparable to that of the radiomics score, and DL does not show its advantages (*P* > 0.05). However, the DL-radiomics score performs best among them (*P* < 0.05), likely due to its combination of low-level (classical radiomics) and high-level (DL) image abstractions for capturing texture patterns (30, 31). Previous studies have similarly confirmed that model trained with multiple types of features exhibit superior performance than any of them alone (32–34).

To improve the predictive performance of CT-based radiomics for GCLM, multivariable logistic regression was used to create a fused model by combining DL-radiomics score and significant clinical factors. Its AUC value was significantly higher than that of all other models (*P* < 0.05) except the DL-radiomics score (*P* > 0.05) [0.796 (95% CI, 0.766-0.826) in the training cohort, 0.787 (95% CI, 0.741-0.834) in the test cohort]. However, it provided a largest clinical net benefit over the relevant threshold range than any other model, indicating that it can make better predictions in various situations. Other models perform well under certain thresholds, but are not as stable as the fused model. At the same time, within a wide range of threshold probability, the fused model consistently outperformed both treat-all and treat-none strategies, suggesting its robustness in balancing overtreatment risks and missed diagnoses. These all substantiated the high predictive accuracy and wide applicability of the fused model, while also demonstrating that the comprehensive inclusion of meaningful features can enhance the model’s ability to learn from a broader dataset, thereby improving its precision, robustness, and generalizability. The calibration curve confirms that the GCLM positive probability value predicted by the fused model is in good agreement with the actual probability value, avoiding the risk of model overfitting (35). Then, we visualized the fused model into a nomogram, which serves as an intuitive tool that provides personalized risk assessments in the form of scores, based on specific clinical factors and imaging data of patients (36). This aids clinician determining the likelihood of LM, enabling early identification of high-risk patients and thereby facilitating the formulation of more appropriate treatment plans.

Furthermore, we validated that DL-radiomics score can be employed to distinguish between patients with synchronous and metachronous GCLM. A number of studies have shown that the overall survival of patients with synchronous GCLM is worse than that of patients with metachronous GCLM (3). In the field of CCLM, many scholars have made studies to prove the difference between the synchronous and metachronous CCLM. They believed that the pathological differences between the two led to the treatment effect and prognosis of synchronous CCLM are worse than those of metachronous CCLM, and synchronous CCLM may be a more invasive disease (37, 38). Therefore, the management of synchronous and asynchronous CCLM needs to be personalized to meet the needs of each patient and achieve better therapeutic effect. Similarly, our results showed that DL-radiomics score has moderate ability to distinguish synchronous GCLM and metachronous GCLM patients. This finding suggests that there are differences between the two types of metastasis at the imaging phenotype level, and its potential biological heterogeneity may result in different overall survival rates.

There are still some limitations in our research. First of all, this is a single-center retrospective study, and no external validation has been established. The limitations of sample sources may affect the representativeness of the model results. Secondly, CT images come from different devices, which may have some minor effects on the results. Finally, our model was only based on CT images in the venous phase, and images in the plain and arterial phases were not included in the study.

## 5 Conclusion

In summary, we developed a CT-based fused model achieved better predictive performance and stability than models based only on clinical factors or one type of radiomics features. The results of model can predict the risk of LM in GC patients. At the same time, the DL-radiomics score combining classical radiomics features and DL features also showed moderate ability to distinguish synchronous GCLM and metachronous GCLM, which provided a reference for personalized follow-up and timely treatment of patients.

## Data Availability

The original contributions presented in the study are included in the article/**Supplementary Material**. Further inquiries can be directed to the corresponding author.
