# Supplementary material for: Combining radiomics and deep learning to predict liver metastasis of gastric cancer on CT image
Source: Front Oncol. 2025 Jun 24;15:1613972. doi: 10.3389/fonc.2025.1613972 (PMC12234316; doi:10.3389/fonc.2025.1613972)
Supplement: Supplementary file 1 [file DataSheet1.docx]

**.Supplementary Methods**

**Appendix S1: CT examinations**

All patients underwent abdominal enhanced CT examination before receiving GC treatment. Scanning methods: patients were fasted for at least 8 hours before scanning. In order to reduce gastrointestinal motility, 10 mg of anisodamine was injected intramuscularly 10-20 minutes before scanning. Before entering the examination room, 1000 ml of water was taken orally to fill the stomach, so as to facilitate the display of lesions. Some patients take in water according to the condition of the disease. The patient was in a supine position to the examination bed. The upper boundary of the scanning range was the diaphragm top, and the lower boundary was to the upper edge of the pubic symphysis. All patients underwent conventional 64-slice CT three-phase enhanced examination. The scanning parameters were: tube voltage 120kV, tube current 240-300mA, and layer thickness 5mm. Non-ionic iodinated contrast agent iohexol (iodine content 350g/L) was injected through the elbow vein at 2.5-4.0 mL/s, and the total dose was calculated as 1.5 mL/kg. Arterial phase scanning was performed at 25-30 s after contrast agent injection, venous phase scanning was performed at 60 s, and delayed phase scanning was performed at 180 s.

**Appendix S2: Feature extraction methodology**

**Classic radiomics feature extraction**

Classic radiomics features were computed from the radiologist-drawn the region of interests(ROIs) using an open-source python package PyRadiomics (1). Detailed calculations of classic radiomics features are described and provided in online documentation of PyRadiomics (https://pyradiomics.readthedocs.io/en/latest/features.html). The resampled voxel sizes were set to 1× 1 × 1 mm³ voxels to standardize the slice thickness. Image intensities were binned by 25 HU and voxel array shift were set on 1000. Segmented voxels were resampled at the range of 50 to 350 HU including the whole tumor and excluding the air and bone tissues. Defined radiomic image features without/after wavelet filtration that described tumor characteristics were extracted. Wavelet filtration filtered original image directionally with x, y and z directions respectively (H: High pass filter, L: Low pass filter), yielding 8 different combinations of decompositions. The extracted radiomics features can be divided into 4 groups: (I) shape features, (II)first-order features, (III) second-order features and (IV) high-order features. Most features defined below were in accord with feature definitions as described by the Imaging Biomarker Standardization Initiative (IBSI).

There are differences in gray value discretization (for the fixed bin size type) and resampling that cannot be corrected by customization settings alone and require replacement by custom functions, which are elaborated in the Pyradiomics documents. It is worth noting that two features available in PyRadiomics are not defined in the IBSI, namely: Total Energy and Standard Deviation. Entropy in Pyradiomicsis defined by IBSI asIntensity Histogram Entropy. Uniformity in Pyradiomics is defined by IBSI asIntensity Histogram Uniformity. Mesh Volume in Pyradiomics is defined as Volume. Voxel Volume in Pyradiomics is defined in IBSI as Approximate Volume. Joint Energy in Pyradiomics is defined by IBSI as Angular Second Moment. Maximum Probability in Pyradiomics is defined by IBSI as Joint maximum Sum of Squares in Pyradiomics is defined by IBSI as Joint Variance. The PyRadiomics kurtosis is not corrected, where IBSI kurtosis is corrected by -3, yielding 0 for normal distributions. All the remaining features correspond to the definitions provided by IBSI.

Here, we describe the radiomic features with definitions or implementation that differ from the IBSI reference document used in our model.

1. shape-based 3D features

In this group of features we included descriptors of the 3-dimensional size and shape of ROI. These features are independent from the gray level intensity distribution in the ROI and are therefore only calculated on the non-derived image and mask.

1. First-order statistics features

First-order statistics describe the distribution of voxel intensities within the image region defined by the mask through commonly used and basic metrics.

1. Second-order features

Second-order features can reflect the homogeneity phenomenon of the images and the arrangement of the properties that change slowly or periodically on the body surface. Textural features extracted in our study included five types of matrix features, including gray-level co-occurrence matrix (GLCM) features, gray-level run length matrix (GLRLM) features, gray-level size zone matrix (GLSZM) features, Neighbouring Gray Tone Difference Matrix (NGTDM), and gray-level dependence matrix (GLDM) features. Determining the texture matrix representations requires the voxel intensity values within the ROI to be discretized.

Voxel intensities were therefore resampled into equally spaced bins using a bin-width of 25 Hounsfield units. This discretization step not only reduces the image noise but also normalizes the intensities across all patients, allowing for a direct comparison of all the calculated textural features between patients.

A GLCM describes the distance and angle of each pixel, which calculates the correlation between two gray levels with certain directions and distances. GLCM can reflect integrated information regarding the direction, interval, amplitude, and frequency of the images. As for GLRLM, the run length metrics quantify the gray level runs in an image. A gray level run is defined as the length in the number of pixels and of the consecutive pixels that have the same gray-level value. A GLSZM describes the amount of homogeneous connected areas within the tumor volume, of a certain size and intensity, thus reflecting the tumor heterogeneity at a regional scale.

1. High-order features

Seven image filters were applied to original image respectively and yield a corresponding derived image.

A Laplace of Gaussian (LoG) spatial band-pass filter was used to derive image features at different spatial scales by turning the filter parameter with 1.0, 3.0 and 5.0. Wavelet transformation effectively decouples the textural information by decomposing the original image in low- and high-frequencies. In our present study, a discrete, one-level and undecimated two-dimensional wavelet transformation was applied to each CT image, which decomposed the original image into 4 decompositions. Consider L and H to be low-pass and high-pass functions, respectively, X to be the decomposing image, and the wavelet decompositions of X to be labeled as XLL, XLH, XHL, XHH. Then, four new images that are decomposed in two directions (x, y) can be obtained. Since the applied wavelet decomposition is undecimated, the size of each decomposition is equal to the original image and each decomposition is shift invariant. Thus, the original tumor delineation of the tumor volume can be applied directly to the decompositions after wavelet transformation. Similarly, square, squareRoot, logarithm, exponential, and gradient filters were also applied to original image respectively and yield a corresponding derived image.

**Deep learning feature extraction**

For the deep learning feature extraction, we first extracted the three-dimensional ROIs from the standardized CT inputs. A two-step process for 3D ROI extraction was implemented in this study: (a) the 3D bounding box tightly around the tumor was calculated according to the radiologist’s annotation; (b) the 3D ROI was extracted from the standardized CT input according to its corresponding 3D bounding box.

Given the 3D ROIs, we formulated the deep learning feature extraction as an unsupervised learning task rather than a supervised learning task. Therefore, we constructed an autoencoder (AE) based on deep convolutional neural network (DCNN) to extract the deep learning feature. The AE consisted of two components: a 3D encoder and a 3D decoder. The 3D encoder was used to extract the latent-space vector from 3D ROIs automatically; Then, the decoder recovered CT slices from the latent-space vector so that the slices were as close as possible to the encoder's input. In this study, the latent-space vector was called the deep learning feature.

The architecture details of our AE were shown in Figure 2. In this study, the 3D encoder consisted of four stacked down-block modules and a linear module. Each down-block module was composed of a 3D convolution (Conv3d) operation, a 3D batch normalization (BN3d), and a leaky rectification linear unit (LeakyReLU). The kernel size of the 3D convolution operation was set to 4 × 4 × 4, the stride was set to 2x2x2, and the padding was set to 1x1x1. The negative slope of LeakyReLU was set to 0.2. The linear module also named the fully-connected (FC) layer, has an input size of 9216 and an output size of 512. In other words, the size of the deep learning feature is 512. Through the module-bymodule extraction of the encoder, it can be said that the deep learning features are abstract expressions of the 3D ROI and can describe the characteristics of 3D ROI. Moreover, the decoder also consisted of a linear module and four stacked up-block modules. The parameters of the linear module were set to 512 × 9216. Each up-block module except up-bloack4 was composed of a 3D transposed convolution (ConvTranspose3d), a BN3d, and a LeakyReLU. The parameters of them were the same as those in the encoder. We implemented the AE according to the above description. Since the goal of AE is to make the output of the decoder as close as possible to the input of the encoder, we chose the mean square error (MSE) loss function to train the AE in an unsupervised learning manner. To make the AE training converge faster, we employed WL 40 HU and WW 200 HU to normalize the standardized input to [-1, 1]. We also applied the hyperbolic tangent (Tanh) function behind the decoder to make the output range between -1 and 1. Then, we resized the size of each 3D ROI (represented by [depth, width, height]) to [16, 96, 96] to meet the requirements of mini-batch training. In this study, each mini-batch contained six 3D ROIs. During training progress, a stochastic gradient descent (SGD) with weight decay and momentum optimizer was used to minimize the loss function. The parameters of it were set with initial learning rate, momentum, and weight decay values of 0.0001, 0.1, and 0.0005, respectively. Cosine annealing with warmup was also used to decay the learning rate of each parameter group. The number of iterations of warmup was set to 10. The maximum number of iterations was set to 60. To reduce the time of the input/output operations, eight separate processes were used to read images from the disk to the memory. The parameters of the model were saved when the models achieved the minimal MSE on the validation subset. Our AE was implemented using the Python Pytorch package (<https://pytorch.org>).

**Appendix S3: Feature selection and scores building**

**Classic radiomics feature selection**

After the variance threshold method, the 1337 features with variance≤1.0 were removed from 1925 classic radiomics features; the remaining 588 classic radiomics features were retained. Spearman correlation analysis was performed to remove 0 features that had an average correlation coefficient greater than 0.7. Then 365 features were selected through t-test. Finally, the 39 potential features were selected after LASSO algorithm.

**Deep learning feature selection**

After the variance threshold method, the 376 features with variance≤1.0 were removed from 512 DL features; the remaining 136 DL features were retained. Spearman correlation analysis was performed to remove 0 features that had an average correlation coefficient greater than 0.7. Then 30 features were selected through t-test. Finally, the 29 potential features were selected after LASSO algorithm.

**Deep radiomics feature selection**

After the variance threshold method, the 1713 features with variance≤1.0 were removed from 2437 DL radiomics features; the remaining 724 DL radiomics features were retained. Spearman correlation analysis was performed to remove 0 features that had an average correlation coefficient greater than 0.7. Then 395 features were selected through t-test. Finally, the 57 potential features were selected after LASSO algorithm.

**Scores building**

Finally, a signature of each patient with LAGC for predicting GCLM was calculated with a linear combination of the final selection of features and multiplied by their normalized coefficients using the multivariable logistic regression model.

Table S1 The clinical characteristics in all patients.

| Characteristics | Non-LM (n=689) | LM (n=312) | P value |
| --- | --- | --- | --- |
| Age (mean ± SD, years) | 58.75±10.29 | 61.02±10.49 | 0.001* |
| Sex, No. (%) |  |  | 0.030* |
| Female | 166 (24.1) | 56 (17.9) |  |
| Male | 523 (75.9) | 256 (82.1) |  |
| Tumor location, No. (%) |  |  | 0.002* |
| Cardia/fundus | 260(37.7) | 89(28.5) |  |
| Body | 121(17.6) | 59(18.9) |  |
| Antrum | 179(26.0) | 75(24.0) |  |
| More than two-thirds of stomach | 129(18.7) | 89(28.5) |  |
| Tumor thickness ± SD (mm) | 16.82±6.33 | 19.51±7.43 | ＜0.001* |
| Clinical T stage, No. (%) |  |  | 0.113 |
| T1 | 62(9.0) | 23(7.4) |  |
| T2 | 124(18.0) | 41(13.1) |  |
| T3 | 317(46.0) | 147(47.1) |  |
| T4 | 186(27.0) | 101(32.4) |  |
| Clinical N stage, No. (%) |  |  | ＜0.001* |
| N0 | 229(33.2) | 61(19.6) |  |
| N1 | 128(18.6) | 85(27.2) |  |
| N2 | 156(22.7) | 99(31.7) |  |
| N3 | 176(25.5) | 67(21.5) |  |
| Degree of differentiation, No. (%) |  |  | 0.154 |
| Un-/poorly differentiated | 373(54.1) | 184(59.0) |  |
| Moderately/highly differentiated | 316(45.9) | 128(41.0) |  |
| Lauren type, No. (%) |  |  | 0.083 |
| Intestinal | 243(35.1) | 127(40.7) |  |
| Diffuse | 221(32.1) | 104(33.3) |  |
| Mixed | 225(32.8) | 81(26.0) |  |
| Her-2 lever, No. (%) |  |  | 0.279 |
| Negative | 299(43.4) | 124(39.7) |  |
| Positive | 390(56.6) | 188(60.3) |  |
| CEA, No. (%) |  |  | ＜0.001* |
| ≤5(Normal) | 559(81.1) | 176(56.4) |  |
| >5(Abnormal) | 130(18.9) | 136(43.6) |  |
| CA199, No. (%) |  |  | ＜0.001* |
| ≤37(Normal) | 593(86.1) | 211(67.6) |  |
| >37(Abnormal) | 96(13.9) | 101(32.4) |  |

LM: liver metastasis; CEA, carcinoembryonic antigen; CA199, Carbohydrate antigen199; *P<0.05.

**Table S2** Comparison of clinical characteristics between training cohort and test cohort

| Characteristics | Training cohort (n=701) | Test cohort (n=300) | P |
| --- | --- | --- | --- |
| Age (mean ± SD, years) | 59.60±10.35 | 59.12±10.54 | 0.503 |
| Sex, No. (%) |  |  | 0.069 |
| Female | 144(20.5) | 78(26.0) |  |
| Male | 557(79.5) | 222(74.0) |  |
| Tumor location, No. (%) |  |  | 0.660 |
| Cardia/fundus | 242(34.5) | 110(36.7) |  |
| Body | 125(17.8) | 55(18.3) |  |
| Antrum | 176(25.1) | 78(26.0) |  |
| More than two-thirds of stomach | 158(22.6) | 57(19.0) |  |
| Tumor thickness [mean ± SD, (mm)] | 17.55±6.46 | 17.91±7.56 | 0.440 |
| Clinical T stage, No. (%) |  |  | 0.347 |
| T1 | 53(7.5) | 32(10.7) |  |
| T2 | 117(16.7) | 48(16.0) |  |
| T3 | 323(46.1) | 141(47.0) |  |
| T4 | 208(29.7) | 79(26.3) |  |
| Clinical N stage, No. (%) |  |  | 0.325 |
| N0 | 197(28.1) | 93(31.0) |  |
| N1 | 154(22.0) | 59(19.6) |  |
| N2 | 187(26.7) | 68(22.7) |  |
| N3 | 163(23.2) | 80(26.7) |  |
| Degree of differentiation, No. (%) |  |  | 0.828 |
| Un-/poorly differentiated | 388(55.3) | 169(56.3) |  |
| Moderately/highly differentiated | 313(44.7) | 131(43.7) |  |
| Lauren type, No. (%) |  |  | 0.649 |
| Intestinal | 259(37.0) | 111(37.0) |  |
| Diffuse | 233(33.2) | 92(30.7) |  |
| Mixed | 209(29.8) | 97(32.3) |  |
| Her-2 lever, No. (%) |  |  | 0.151 |
| Negative | 307(43.8) | 116(38.7) |  |
| Positive | 394(56.2) | 184(61.3) |  |
| CEA, No. (%) |  |  | 0.664 |
| ≤5(Normal) | 518(73.9) | 217(72.3) |  |
| >5(Abnormal) | 183(26.1) | 83(27.7) |  |
| CA199, No. (%) |  |  | 0.670 |
| ≤37(Normal) | 566(80.7) | 238(79.3) |  |
| >37(Abnormal) | 135(19.3) | 62(20.7) |  |

LM: liver metastasis; CEA, carcinoembryonic antigen; CA199, Carbohydrate antigen199.

Table S3 Uni- and multivariable logistic regression analysis of predictors of GCLM.

|  | **Univariable Analysis** | | **Multivariable Analysis** | |
| --- | --- | --- | --- | --- |
| **Variable** | **Odds Ratio**  **(95% CI)** | **P value** | **Odds Ratio**  **(95% CI)** | **P value** |
| **Tumor location** | 1.26 (1.12-1.41) | ＜0.001* | 1.03 (0.90-1.19) | 0.64 |
| **Tumor thickness** | 1.06 (1.04-1.08) | ＜0.001* | 1.02 (1.00-1.05) | 0.04* |
| **Clinical N-stage** | 1.12 (0.99-1.26) | 0.06 |  |  |
| **CEA** | 3.30 (2.46-4.43) | ＜0.001* | 1.89 (1.30-2.75) | 0.001* |
| **CA199** | 2.93 (2.12-4.04) | ＜0.001* | 1.63 (1.09-2.42) | 0.016* |
| **Radiomics score** | 1635.09 (367.73-8025.45) | ＜0.001* | 0.10 (0.00-8.78) | 0.317 |
| **DL score** | 292.58 (97.94-913.69) | ＜0.001* | 0.43 (0.02-11.50) | 0.616 |
| **DL-radiomics score** | 3241.07 (891.98-12723.78) | ＜0.001* | 10441.15(59.90-2101891) | 0.001* |

GCLM, gastric cancer liver metastasis; CEA, carcinoembryonic antigen; CA199, Carbohydrate antigen199; *P<0.05.

Table S4 The performance of different scores in distinguishing synchronous GCLM from metachronous GCLM.

| **Model** | **AUC** | **ACC** | **SENS** | **SPEC** | **PPV** | **NPV** |
| --- | --- | --- | --- | --- | --- | --- |
| **Radiomics score** | .643 (.590, .696) | .630 (.577, .684) | .575 (.487, .663) | .665 (.598, .732) | .519 (.434, .604) | .713 (.647, .780) |
| **DL score** | .531 (.476, .587) | .547 (.491, .602) | .558 (.469, .647) | .539 (.469, .610) | .432 (.354, .510) | .660 (.586, .735) |
| **DL-radiomics score** | .665  (.613, .718) | .608 (.553, .662) | .725 (.645, .805) | .534 (.463, .605) | .494 (.420, .568) | .756 (.683, .828) |

GCLM, gastric cancer liver metastases ACC, accuracy; SENS: sensitivity; SPEC, specificity; PPV, positive predictive value; NPV, negative predictive value.

| 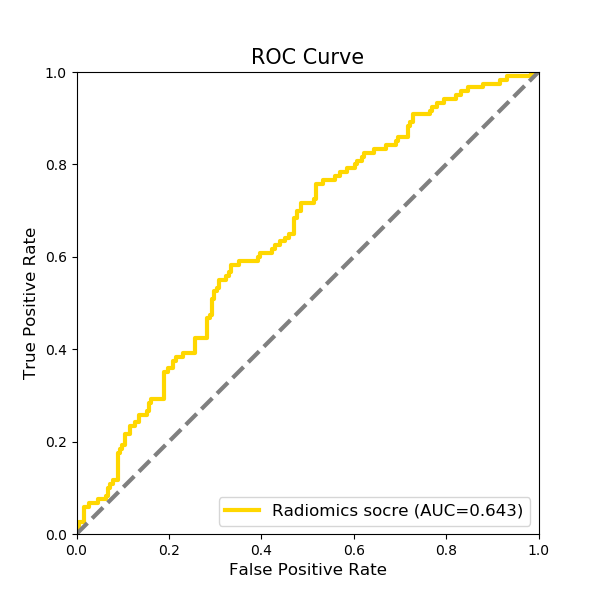a | 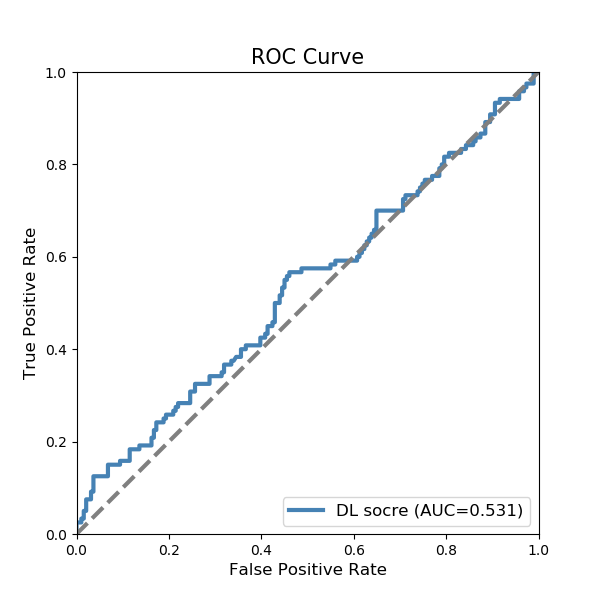b |
| --- | --- |
| c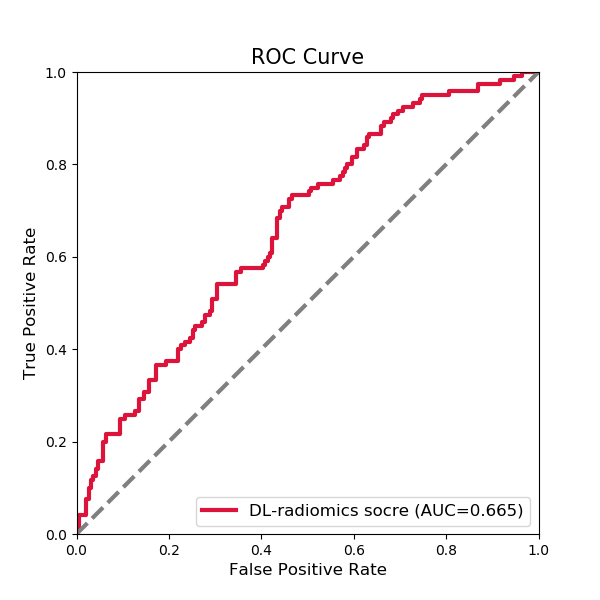 | 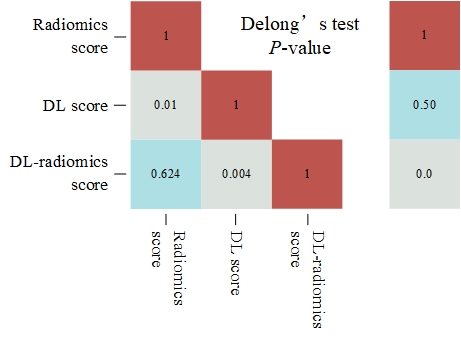d |

Fig S1 ROC curves of different scores to the ability to distinguish synchronous and metachronous GCLM, (a) classical radiomics score, (b) DL score and (c) DL-radiomics score; the heat map shows that the DeLong test compares the statistical results of the AUC values of different models (d). ROC, receiver operator characteristic; GCLM: gastric cancer liver metastases.
